# Supplementary material for: Correlations Between Amelioration of Rotenone-Induced Parkinson’s Symptoms by Amomum tsaoko Flavonoids and Gut Microbiota in Mice
Source: Int J Mol Sci. 2025 Feb 16;26(4):1676. doi: 10.3390/ijms26041676 (PMC11855768; doi:10.3390/ijms26041676)
Supplement: Supplementary file 1 [file ijms-26-01676-s001.zip › ijms-3461360-supplementary.pdf]

Correlations Between Amelioration of Rotenone-Induced Parkinson's Symptoms by *Amomum tsaoko* Flavonoids and Gut Microbiota in Mice

Li Liu [1,2,3,†], Yan Zhao [4,†], Weixing Yang [2], Lixiang Han [2], Xiaohan Mo [2], Jun Sheng [1], Yang Tian [1,*] and Xiaoyu Gao [1,2,3,*]

[1] Yunnan Key Laboratory of Precision Nutrition and Personalized Food Manufacturing, Yunnan Agricultural University, Kunming 650201, China; 2022210085@stu.ynau.edu.cn (L.L.); shengj@ynau.edu.cn (J.S.)
[2] College of Food Science and Technology, Yunnan Agricultural University, Kunming 650201, China; 2021110006@stu.ynau.edu.cn (W.Y.); 15368025745@163.com (L.H.); 15187349230@163.com (X.M.)
[3] Engineering Research Center of Development and Utilization of Food and Drug Homologous Resources, Ministry of Education, Yunnan Agricultural University, Kunming 650201, China
[4] Division of Science and Technology, Yunnan Agricultural University, Kunming 650201, China; 2021013@ynau.edu.cn
* Correspondence: tianyang@ynau.edu.cn (Y.T.); 2018014@ynau.edu.cn (X.G.)
† These authors contributed equally to this work.

Supplementary Table S1. Chemical compounds of ATF (relative abundance >1%, based on plant-widely targeted metabolomics)

NO.	CAS	FORMULA	NAME	CLASS	Relative Abundance (%)
1	35323-91-2	C15H14O6	(+)-Epicatechin	Flavonoids	24.94%
2	482-35-9	C21H20O12	Isoquercitrin	Flavonoids	14.02%
3	480-10-4	C21H20O11	Astragalin	Flavonoids	7.66%
4	17650-84-9	C27H30O15	Kaempferol-3-O-rutinoside	Flavonoids	6.84%
5	29106-49-8	C30H26O12	Procyanidin B2	Flavonoids	5.51%
6	121-33-5	C8H8O3	Vanillin	Phenols	5.51%
7	153-18-4	C27H30O16	Rutin	Flavonoids	4.57%
8	490-46-0	C15H14O6	L-Epicatechin	Flavonoids	2.99%
9	21293-29-8	C15H20O4	(+)-Abscisic acid	Phytohormone	2.47%
10	24808-04-6	C15H14O5	(-)-Epiafzelechin	Flavonoids	2.05%
11	18829-70-4	C15H14O6	Cianidanol	Flavonoids	1.77%
12	28338-59-2	C27H30O15	Cyanidin 3-rutinoside	Flavonoids	1.66%
13	20312-36-1	C9H10O3	L-3-Phenyllactic acid	Phenylpropanoic acids	1.59%
14	1621-84-7	C15H10O7	Tricetin	Flavonoids	1.46%
15	604-80-8	C28H32O16	Narcissoside	Flavonoids	1.41%
16	4540-25-4	C16H21NO4	Phyllalbine	Alkaloids	1.18%
17	65-85-0	C7H6O2	Benzoic acid	Phenols	1.07%
18	572-30-5	C20H18O11	Avicularin	Flavonoids	1.05%
19	5950-12-9	C16H19NO3	Piperlonguminine	Alkaloids	1.05%

Supplementary Table S2. Primer sequences used for the determination of functional gene expression

Gene Name	Forward Sequence	Reverse Sequence
Muc-2	ATGCCCACCTCCTCAAAGAC	GTAGTTTCCGTTGGAACAGTGAA
ZO-1	GCCGCTAAGAGCACAGCAA	TCCCCACTCTGAAAATGAGGA
Occludin	ATGTCCGGCCGATGCTCTC	TTTGGCTGCTCTTGGGTCTGTAT
Claudin4	GCAGAGCACAGGTCAGATGCA	AGGGCAGGTCCTGGAGAATGT
Claudin3	GCACCCACCAAGATCCTCTATTC	CTGTCTGYCCTCTTCCAGCCTAG
COX-2	AACATTCCTTCCCCCAGCAA	TCTATCACTGGCATCCGCTG
IL-1β	TCCATGAGCTTTGTACAAGGA	AGCCCATACTTTAGGAAGACA
TNF-α	AGACCCTCACACTCAGATCA	TCTTTGAGATCCATGCCGTTG
IL-6	CTGCAAGAGACTTCCATCCAG	AGTGGTATAGACAGGTCTGTTGG
MCP-1	TTAAAAACCTGGATCGGAACCAA	GCATTAGCTTCAGATTTACGGGT

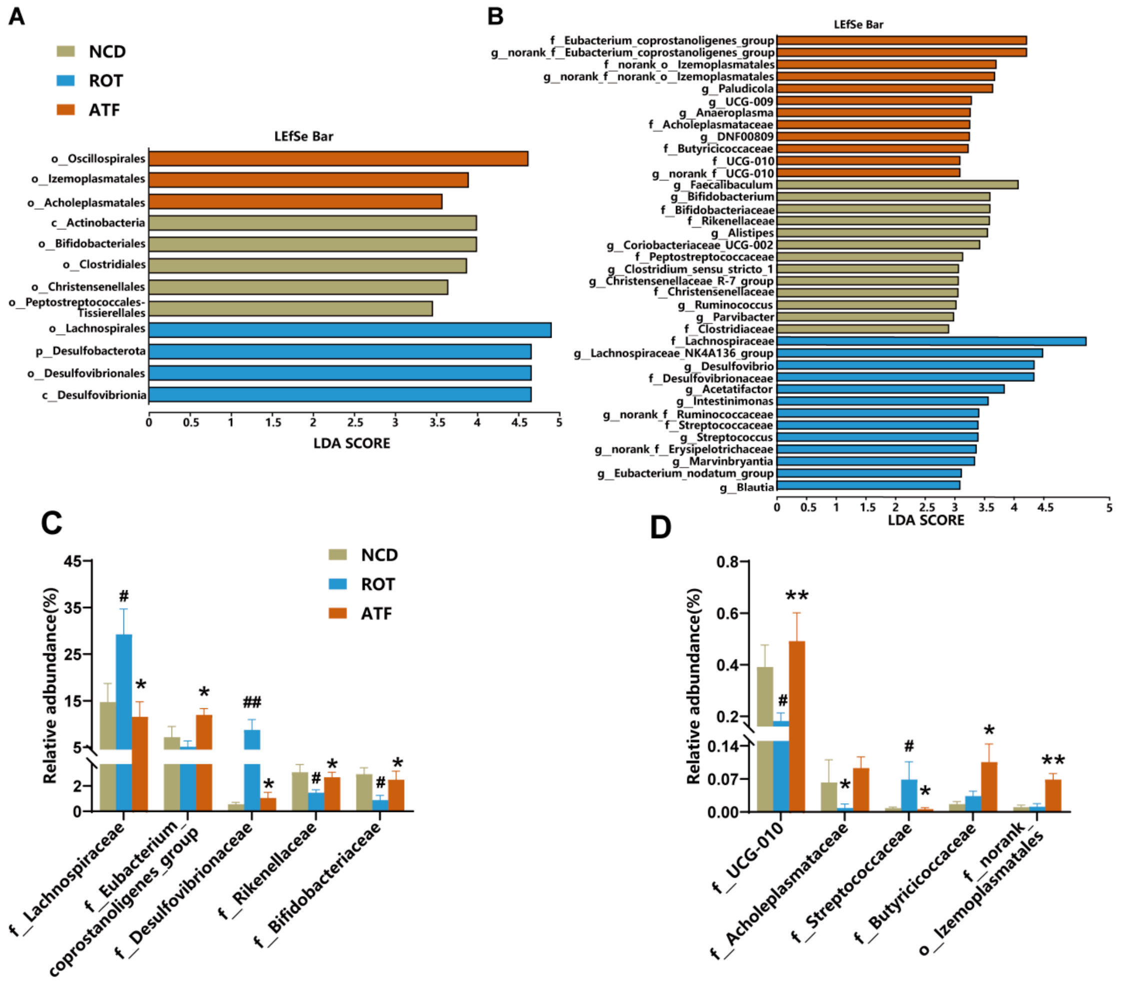

Figure S1. The effect of ATF on the gut microbial composition in PD mice

A-B. Linear discriminant analysis (LDA) of three groups (LDA score>2.0); C-D. The relative abundance of microbes at the family level.
